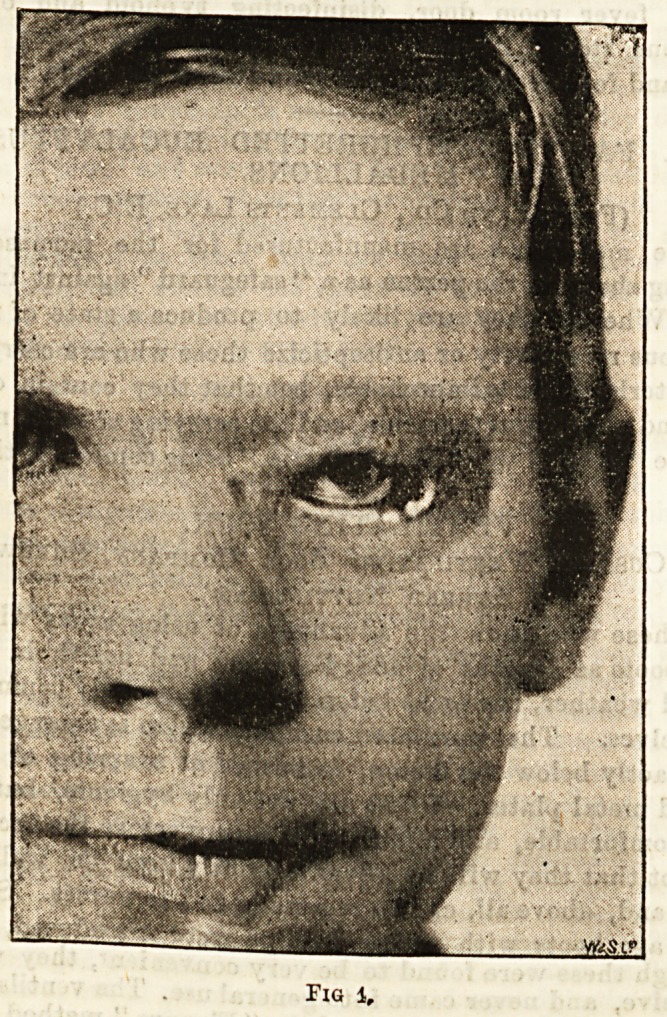# The Treatment of Cosmetic Defects.—I

**Published:** 1893-06-17

**Authors:** 


### BATH EYE INFIRMARY.

### The Treatment of Cosmetic Defects.—I.

In no class of disease are defects more disfiguring than in those affecting the visible parts of the eyes and eyelids. They are always *en evidence*, and cannot be covered, as those of the ears for instance, by arranging the hair, or those of other parts of the body by clothing. Consequently the ophthalmic, perhaps more than other surgeons, is called upon very frequently to neutralise by art the numerous congenital and pathological conditions which, although they may not be the cause of pain or inconvenience, yet, from their conspicuous situation, are a very real annoyance to the patient. Fortunately in the large number of deformities and diseases, both acquired and congenital, which distort or disfigure the eye, something can be done by surgical art to remedy, or at least to render them less objectionable. To mention only a few: The lids may be distorted by coloboma, or disfigured by so slight a thing as a chalazion, a nævus, or a wart. Or the lids may be more or less everted, exposing a constantly red and moist conjunctiva unpleasant to behold as well as annoying to the sufferer. The lids may droop as in ptosis, or be united together as in ankyloblepharon. The cornea may be opacified by leucoma, disfigured by pterygium, distorted by anterior staphyloma, or tied to the lids by symblepharon. The walls of the orbit may be nodulated by exostoses, and imperfect action of the muscles may show itself in squint. Legion is the only inclusive name that sums up the hydra-headed deformations of the ocular and its accessory apparatus. But, fortunately, though the ill-favoured appearance caused by these deformities is so conspicuous, yet it is in most cases amenable to treatment, and the surgeon will often earn more gratitude for a successful cosmetic operation than he will for many a more important one in which, perhaps, life itself is the stake.

Fig. 1.

It is not, however, vanity alone which prompts these patients to come to the surgeon. With many of them it is a question of earning their daily bread. Take, for instance, a very common defect like squint. There are here, of course, other beside the cosmetic reasons for remedying the dissight; but even if there were not, yet

Fig. 2.

with a large number of persons, who have to gain a living in a subordinate position, it is absolutely necessary for them either to change their occupation, or to have the strabismus cured. No man with a squinting eye could hope to get employment in the army, in the navy, or in the police; he would be unfitted for an actor, and he would not be accepted as an assistant in any first or second rate shop, and very few would take him as a clerk. For a woman, squint is a still more serious disability, for with her, whether in the matrimonial or any other market, good looks are never a negligible quantity, and there are few occupations open to the sex in which appearance counts for nothing.

A few cases from the books of the Bath Eye Infirmary will illustrate the points touched upon. A. B., æt. ten years, had suffered from a neglected ophthalmia neonatorum soon after birth which destroyed the function of the right eye. Fortunately no staphylomatous condition ensued, but the cornea was marred by a dense leucoma, very conspicuous and very disfiguring. The condition is shown in Fig. 1, taken from a photograph. It will be observed that there is no margin of iris visible, so that an iridectomy for optical purposes was quite out of the question. Nor, after so long a lapse of time, was it possible for any clearing of the cornea to take place, even if there had been any perception of light, which there was not. The friends were naturally very much distressed, and wished the eye removed and replaced by a glass one. But as a blind eye that is not irritable is preferable to an artificial one, it was resolved to try the effect of tattooing the cornea in the usual manner to resemble the pupil. The eye having been anæsthetised with a 10 per cent. solution of cocaine, a number of pricks into the stroma of the cornea were made by means of a bundle of very fine sewing needles, taking care to make the pricks only in that portion of the cornea which corresponded to the part in the other eye that covered the pupil. A thick paste of Indian ink was then rubbed into the minute punctures by means of a small vulcanite spatula. Care has to be taken in these simple operations not to use pointed fixation forceps to steady the eye, or the result

FIG. 3.

may be that there is small patch of pigment in the conjunctiva where of course it is not wanted. An interval of a few weeks should elapse for any slight

FIG 4.

conjunctival irritation to subside and then tattooing may be repeated. Usually half-a-dozen or more sittings are required before an artistic result is obtained.

Fig. 2 shows the same patient as Fig. 1 after four operations, and already a marked improvement in the cosmetic effect is produced.

Fig. 3 is a case of ankyloblepharon in which the palpebral orifice was much contracted subsequent to a severe burn by molten metal. The boy complained of a constricted feeling, and the usefulness of the eye was much impaired by his inability to raise the upper lid sufficiently to uncover his visual axis. A silver wire was passed through the hypertrophied skin, cellular tissue and conjunctiva, and the ends were bent down over the cheek. It was allowed to remain for two months in order that the edges of the sinus might heal, in the same way that punctures for earrings heal, and then an incision was made from the puncture through the tissues horizontally outwards. It would be useless in such a case to divide the tissues without as a preliminary inserting a wire as such a wound would heal from the angle very rapidly and the resulting cicatrix would only increase the contraction and intensify the disfigurement. Fig. 4 shows this case three months later, when the wire had been cut out, and although the result does not show much improvement in the figure, yet as more of the cornea and pupil were distinctly visible, the cosmetic appearance was decidedly heightened. Another and more important result was that his line of vision horizontally was no longer interfered with in looking straight in front of him. The eye, in fact, instead of as formerly being only useful to him in looking down could now be used in walking.

*(To be continued.)*

## Figures and Tables

**Fig. 1. f1:**
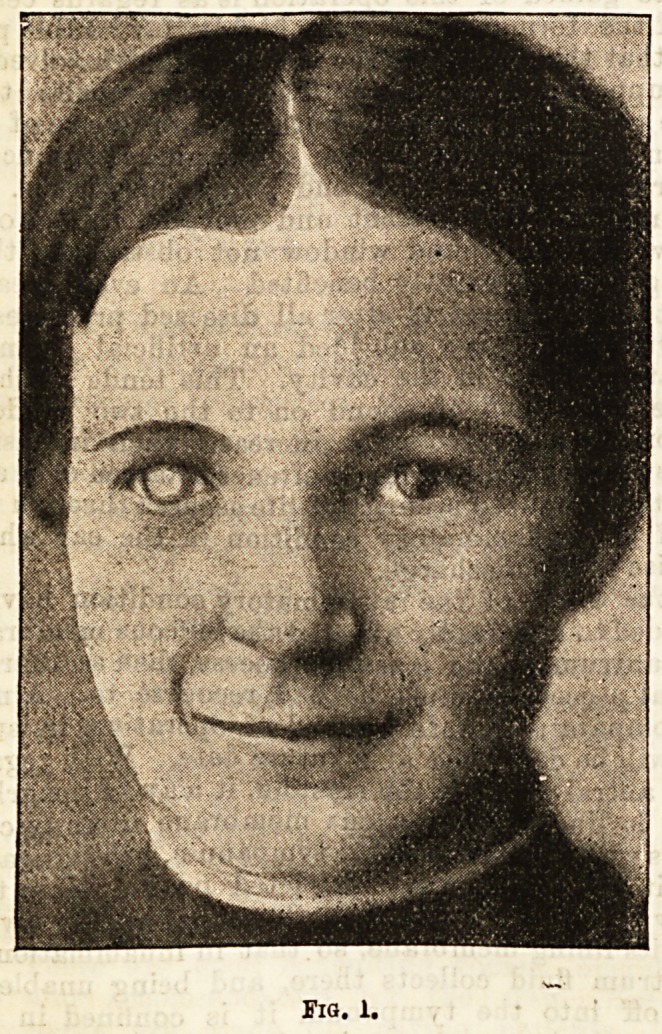


**Fig. 2. f2:**
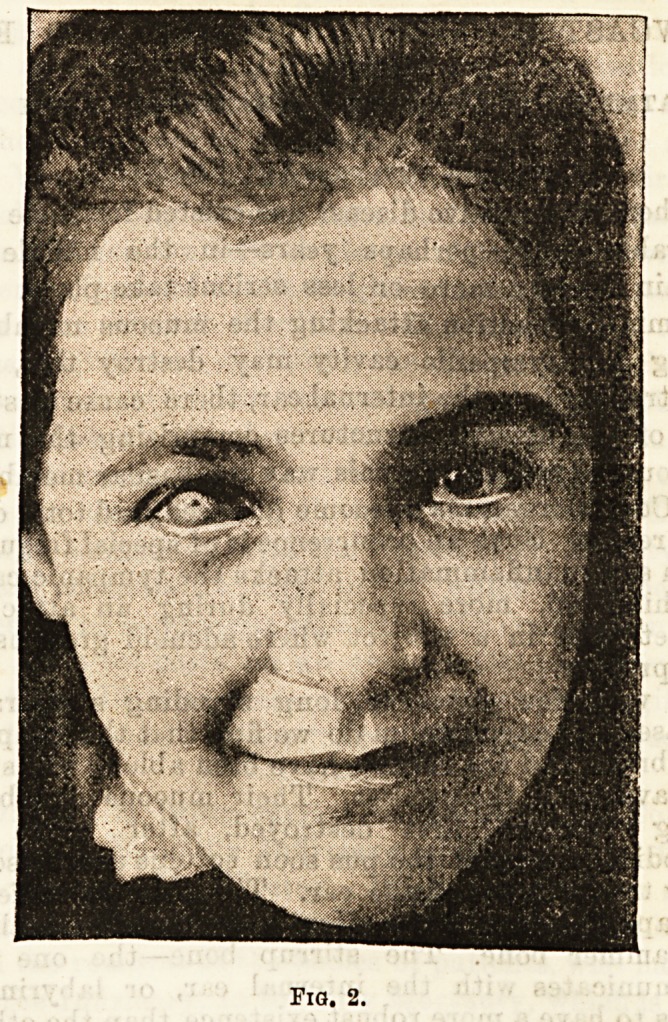


**Fig. 3. f3:**
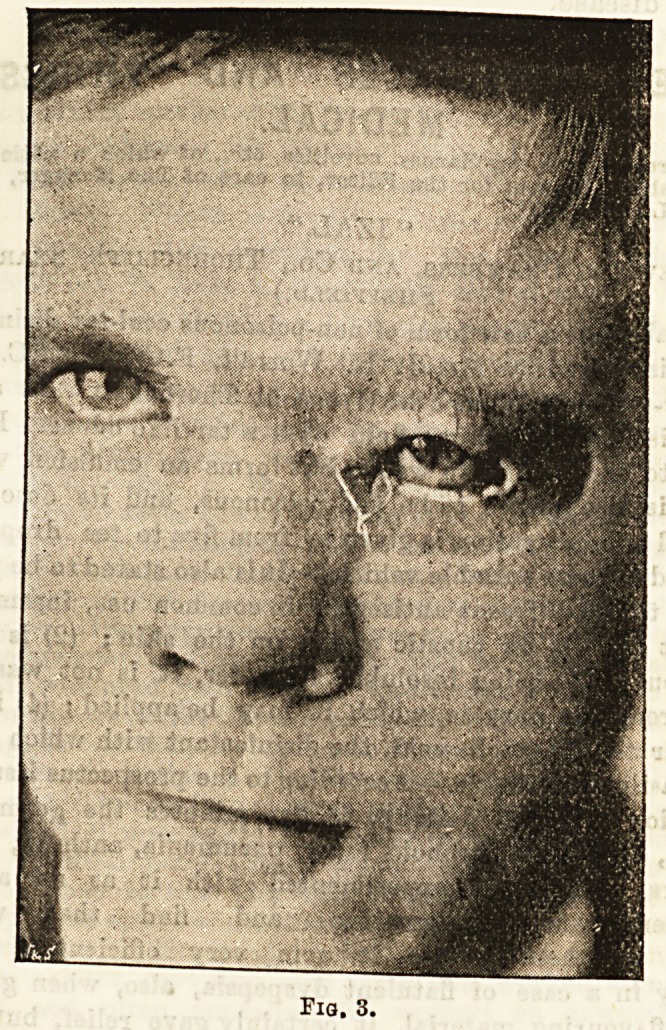


**Fig 4. f4:**